# Therapy‐related chronic myeloid leukemia in a patient receiving peptide receptor radionuclide therapy for pancreatic neuroendocrine tumor

**DOI:** 10.1002/cnr2.1282

**Published:** 2020-09-07

**Authors:** Selin Kucukyurt, Yeliz Yagiz Ozogul, Abdulkadir Ercaliskan, Levent Kabasakal, Ahmet Emre Eskazan

**Affiliations:** ^1^ Division of Hematology, Department of Internal Medicine, Cerrahpasa Faculty of Medicine Istanbul University‐Cerrahpasa Istanbul Turkey; ^2^ Department of Internal Medicine, Cerrahpasa Faculty of Medicine Istanbul University‐Cerrahpasa Istanbul Turkey; ^3^ Department of Nuclear Medicine, Cerrahpasa Faculty of Medicine Istanbul University‐Cerrahpasa Istanbul Turkey

**Keywords:** chronic myeloid leukemia, neuroendocrine tumor, peptide receptor radionuclide therapy, therapy‐related leukemia, tyrosine kinase inhibitor

## Abstract

**Background:**

Therapy‐related leukemia is a well‐recognized clinical syndrome. Peptide receptor radionuclide therapy (PRRT) is a modern therapeutic approach using radionuclide combined with somatostatin analog peptide for inoperable or metastatic neuroendocrine tumors.

**Aims:**

Hematologic toxicities including late‐onset myeloid neoplasms have been reported after PRRT; however, therapy‐related chronic myeloid leukemia (TR‐CML) following PRRT is a relatively rare entity.

**Methods:**

We present a 64‐year‐old male who received PRRT for pancreas neuroendocrine tumor and then developed TR‐CML 60 months after the initiation of PRRT. The patient responded well to imatinib therapy.

**Results:**

Patients with TR‐CML generally have similar tyrosine kinase inhibitor responses and outcomes when compared to de novo cases.

**Conclusions:**

The physicians should be aware of the short‐ and long‐term hematologic toxicities of PRRT including TR‐CML, and careful monitoring is mandatory in this group of patients.

## 1 INTRODUCTION

Chronic myeloid leukemia (CML) is a myeloproliferative neoplasm characterized by the overproduction of myeloid cells and the presence of Philadelphia (Ph) chromosome that is characterized by the reciprocal translocation of chromosomes 9 and 22—t(9;22). Risk factors for CML include older age, male gender, and radiation exposure.^1^, ^2^, ^3^

Therapy‐related leukemia (TR‐L) is defined as having a prior history of any malignant neoplasm that was treated with chemotherapy and/or radiotherapy prior to the time of diagnosis of leukemia.^4^ On the other hand, therapy‐related CML (TR‐CML) is a relatively rare entity, and approximately, 150 cases of TR‐CML or CML as a secondary malignancy have been reported in the literature.^4^, ^5^, ^6^, ^7^

TR‐L can be associated with certain karyotypes such as abnormalities of chromosomes 5 and/or 7 or a complex karyotype.^5^ On the contrary, isolated t(9;22) (q34;q11) abnormalities occur in TR‐CML, and additional cytogenetic abnormalities are not usually observed.^6^ However, Kantarjian et al^5^ have detected monosomy 7 in addition to t(9;22) in a patient who was diagnosed with Ph + CML at 126th month of cytotoxic chemotherapy for primary ovarian cancer.

Gastroenteropancreatic neuroendocrine tumors (GEP‐NETs) constitute a heterogenous group of tumors with their origin in neuroendocrine cells, and most commonly, the primary lesion is located in gastric mucosa, intestine, rectum, and pancreas. Peptide receptor radionuclide therapy (PRRT), which is a tumor‐targeted strategy that uses radiation to induce tumor cell death in NET via β particle–emitting radionuclide radiolabeled to a somatostatin peptide analog.^8^ PRRT with ^177^Lu‐DOTA^0^‐Tyr^3^‐octreotate (^177^Lu‐DOTATATE) or ^*90*^ *Y*‐DOTA^0^‐Tyr^3^‐*octreotide* (^*90*^ *Y‐DOTATOC*) is an effective therapeutic approach in the treatment of inoperable or metastatic GEP‐NETs that express somatostatin receptors.^9^ Sunitinib is a multitarget tyrosine kinase inhibitor (TKI), which is one of the treatment options in patients with advanced pancreatic neuroendocrine tumors (PNETs).^10^ We herein present a case who was first diagnosed with PNET and received sunitinib and PRRT and then developed TR‐CML during the follow‐up.

## 2 CASE REPORT

A 64‐year‐old male patient was diagnosed with PNET in November 2012. The initial complete blood count revealed a leukocyte count of 9.6 × 10^9^/L (neutrophils, 63.8%; lymphocytes, 28%; monocytes, 6.9%; and basophils, 0.5%), hemoglobin 15.6 g/dL, and platelet count of 244 × 10^9^/L. In February 2013, the patient underwent a distal pancreatectomy and splenectomy. Multiple focal lesions of liver were detected following surgery, and then he started sunitinib together with long‐acting somatostatin analogue (Sandostatin LAR 20 mg, Novartis) in March 2013. Following splenectomy, leukocyte count was 9.4 × 10^9^/L, hemoglobin was 14.3 g/dL, and platelet count was 427 × 10^9^/L. Six weeks later, he had to stop sunitinib permanently due to uncontrolled hypertension and skin toxicity.

Abdominal magnetic resonance imaging and Ga‐68‐Dota‐Tate PET/CT revealed liver metastases. ^177^Lu‐based PRRT was started in February 2014 and a dose of 3.7 GBq was given per cycle. After four doses, in August 2015, the patient received 1.35 GBq of trans‐arterial Y‐90‐Microsphere treatment for the right liver lobe. ^117^Lu‐DOTATATE treatment was administered for six cycles, approximately 12 weeks apart, with 3.7 GBq infused at each cycle with a cumulative dose of 33.3 GBq (900 mci) until October 2018. Then, ^117^Lu‐DOTATATE treatment was *interrupted due to* refractory disease.

**FIGURE 1:**
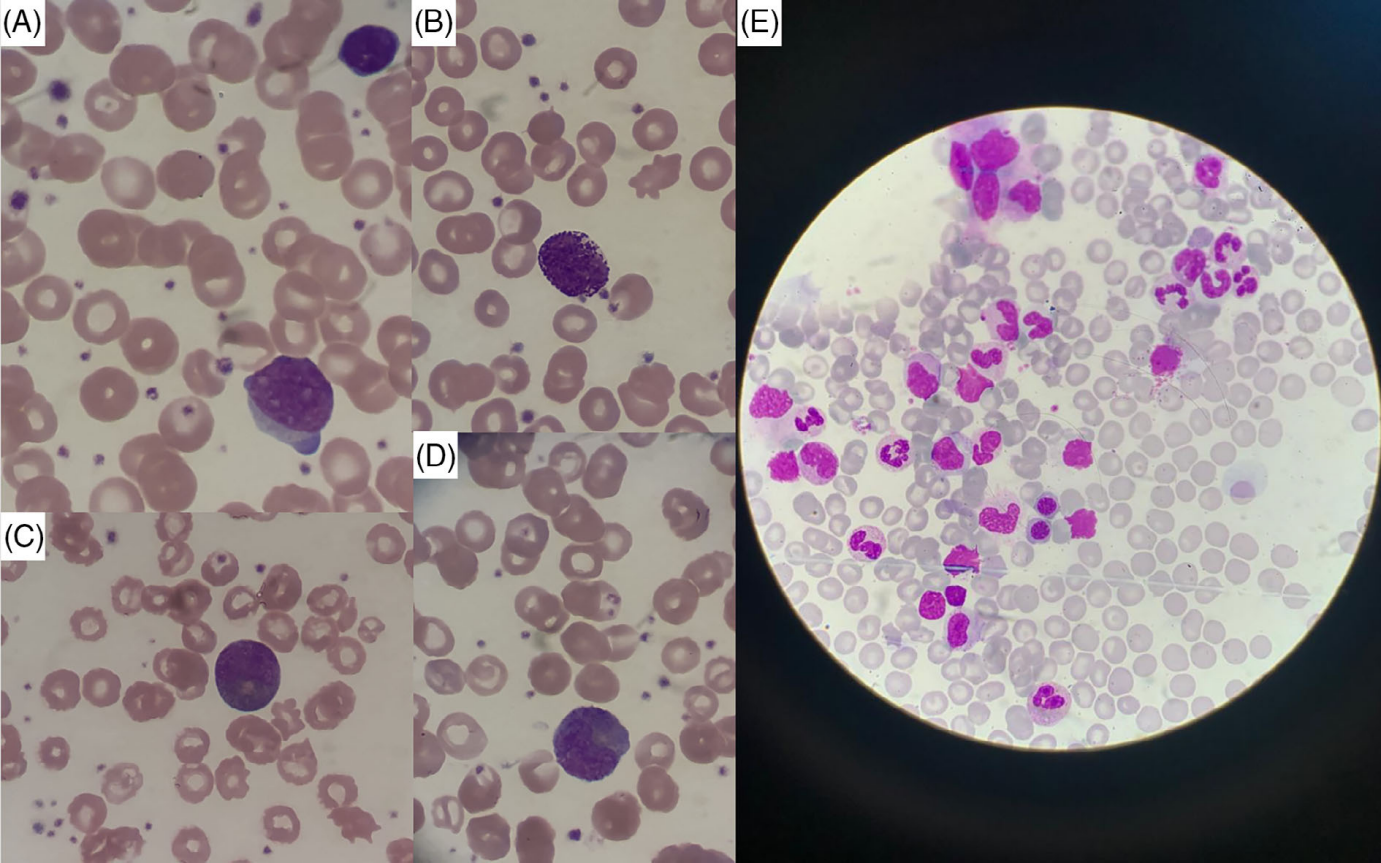
The peripheral blood and bone marrow aspirate smears of our patient. A, Blood film reveals a blast, lymphocyte, and anisothrombi (Wright‐Giemsa×1000). B, Blood film shows normal‐appearing basophil. C, Blood film reveals a myelocyte. D, Blood film shows a metamyelocyte. E, Bone marrow aspirate shows hyperplasia of elements of the granulocytic series, also polychromatophilic normoblasts, and blue sea histiocyte

During the follow‐up, the patient had persisting leukocytosis and thrombocytosis, which were thought to be due to postsplenectomy state. The patient was evaluated because of progressive neutrophilic leukocytosis in February 2019. He had no complaints or showed no signs/symptoms of infection. Leukocyte count was 42.1 × 10^9^/L, hemoglobin was 11.7 g/dL, and platelet count was 692 × 10^9^/L. Peripheral blood smear showed granulocytosis with different stages of maturation from myelocyte to neutrophils; 5% myelocytes, 2% metamyelocytes, 6% band cells, 59% neutrophils, 10% basophils, 2% eosinophils, 13% lymphocyte, and 3% monocyte. Peripheral blood and bone marrow aspirate smears at the time of diagnosis are shown in Figure 1.

CML was confirmed by interphase fluorescence in situ hybridization (FISH) for t(9;22) from the peripheral blood, which was 51.2% positive. At diagnosis, the bone marrow aspirates showed hypercellular marrow with marked myeloid hyperplasia and increased megakaryocytes. In conventional cytogenetic evaluation of bone marrow, no metaphases were found for analysis. Due to splenectomy, disease risk scores could not be calculated. p210 *BCR‐ABL1* was detected as 55.49% in the peripheral blood, and imatinib 400 mg/day was started in March 2019. At the first month of the therapy, complete hematologic response (CHR) was obtained, and *BCR‐ABL1* ^*IS*^ was 0.21% at 3 months of TKI therapy.

At the sixth month of imatinib in September 2019, bone marrow cytogenetics showed 46, XY,^11^ and tetraploid,^1^ with no Ph + metaphases. Also, bone marrow FISH was negative for t(9;22), and *BCR‐ABL1* ^*IS*^ was 0.0182%, thus complete cytogenetic and major molecular responses (MMRs) were achieved.

**FIGURE 2:**
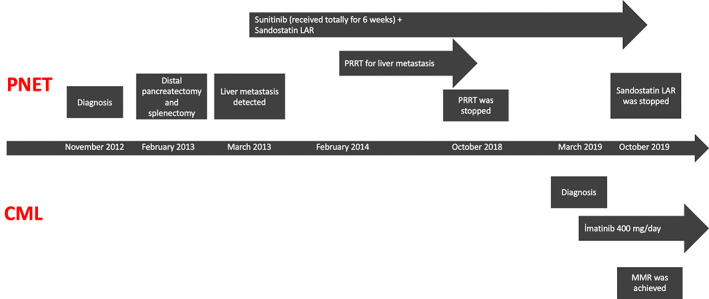
Treatment timeline of our patient (CML, chronic myeloid leukemia; MMR, major molecular response; PNET, pancreatic neuroendocrine tumor; PRRT, peptide receptor radionuclide therapy)

For PNET, the patient continued receiving octreotide until October 2019, and this treatment was discontinued subsequently due to refractory disease. Transarterial chemoembolization was planned, but the patient refused this treatment modality. The timeline of treatment sequence was displayed in Figure 2.

Then the patient discontinued imatinib for 1.5 months due to nausea and vomiting, and at the 9th month of TKI therapy in December 2019, MMR was lost (*BCR‐ABL1* ^*IS*^ was 2.54%). We rechallenged imatinib with antiemetics, which was well tolerated. Molecular response status has not been evaluated yet due to COVID‐19 pandemic.

## 3 DISCUSSION

Patients with TR‐L are typically at high risk, and their outcomes are inferior than those with the corresponding de novo cases.^5^ However, prior to TKI era, the treatment responses were shown to be generally similar in both cases with TR‐CML and de novo CML.^4^, ^6^ Similarly, in the TKI era, response to TKI treatment is excellent in patients with TR‐CML in chronic phase.^6^, ^11^ Iriyama et al^6^ have investigated the prognosis of 297 de novo CML and 11 TR‐CML patients with a median follow‐up of 48 months. Primary malignancy was solid tumor in 10 patients, while it was acute lymphoblastic leukemia in one patient. Eight patients had a history of chemotherapy, two had radiotherapy, and one patient received both. The median time to TR‐CML diagnosis was 7 years. All patients with TR‐CML achieved optimal responses, and there was no significant difference between TR‐CML and de novo CML cases in terms of responses and event‐free survival. Our patient also responded well to TKI treatment, and MMR was achieved at the 6th month of imatinib.

Sunitinib can be associated with multiple toxicities, and cases who developed CML following sunitinib use have been described in the literature.^10^ However, these cases have long‐term treatment history with sunitinib, unlike our case. Systemic treatment options for advanced GEP‐NETs include PRRT. Subacute bone marrow toxicities, which include thrombocytopenia, anemia, leukopenia, and lymphopenia, are the main dose limiting hematologic adverse events of PRRT. Nadir counts commonly occur 4 to 6 weeks and improve within 8 weeks.^12^ The most serious long‐term toxicity associated with PRRT is irreversible myelotoxicity. Late‐onset myeloid neoplasms have been reported after treatment with PRRT with a mean incidence of 2.6%,^8^ however, a few cases of TR‐CML following PRRT were reported.^8^, ^13^, ^14^, ^15^

Brieau et al^16^ followed 20 patients treated with ^177^Lu‐octreotate for NET with a median of 3.1 years, and four cases developed myelodysplastic syndrome (MDS) or acute myeloid leukemia (AML) in 30, 31, 54, and 70 months after the first cycle of PRRT. However, all patients had previously received a chemotherapy containing an alkylating agent. Bergsma et al^13^ reported the prevalence of persistent hematologic dysfunction (PHD) as 4% (11/274) after PRRT with ^177^Lu‐DOTATATE in 274 cases with GEP‐NET. The median time from first PRRT cycle to PHD diagnosis was 41 months. In this case series, only one patient developed TR‐CML 42.3 months following PRRT. This patient was a 61‐year‐old male with history of prior chemoembolization, and he was Ph + with no additional cytogenetic abnormalities. Our case did not receive any chemotherapy, and he developed TR‐CML 60 months after the initiation of PRRT.

Clonal chromosomal abnormalities in the Ph‐negative (CCA/Ph−) metaphases after TKI treatment can be observed in patients with CML.^17^ In our case, no metaphases were detected initially, and thus, we cannot spaculate whether the tetraploidy was present at the time of CML diagnosis or not. In addition to that, tetraploidy was detected in only one metaphase, and CCAs are generally defined as abnormalities present in ≥2/20 metaphases or if the abnormalities are present in one metaphase in ≥2 assessments.^17^ So, since we did not perform another bone marrow cytogenetic evaluation yet, we cannot say this cytogenetic abnormality is clonal, and also we cannot comment on the possible impact of this tetraploidy on the outcome of our patient with a relatively short follow‐up period under TKI therapy.

In conclusion, although TR‐myeloid neoplasms can be observed in patients receiving PRRT, TR‐CML is a rare event following PRRT. TR‐AML and TR‐MDS patients generally have inferior prognosis than that of de novo cases; on the other hand, patients with TR‐CML generally have similar TKI responses and outcomes when compared to de novo patients. Although our patient is an optimal responder to TKI therapy, the follow‐up duration is relatively short, thus, we cannot comment on the long‐term outcome of the defined case. PRRT is an effective and relatively safe treatment option; however, physicians should be aware of the long‐term hematological toxicities of this treatment modality including TR‐CML.

## CONFLICT OF INTEREST

Ahmet Emre Eskazan has received advisory board honorarium from Novartis, and he also received speaker bureau honoraria from Novartis and Bristol‐Myers Squibb and Pfizer outside the present study. Other authors have no conflicts of interest to declare.

## AUTHORS' CONTRIBUTIONS

All authors had full access to the data in the study and take responsibility for the integrity of the data and the accuracy of the data analysis. *Conceptualization*, S.K., Y.Y.O, A.E., A.E.E.; *Data Curation*, S.K.,Y.Y.O, A.E., L.K., A.E.E.; *Formal Analysis*, S.K., L.K., A.E.E.; *Investigation*, S.K., Y.Y.O., A.E., L.K., A.E.E.; *Methodology*, S.K., Y.O.O, A.E.E.; *Resources*, S.K., Y.Y.O., L.K., A.E.E; *Software*, S.K., Y.Y.O, A.E.E; *Visualization*, S.K.; *Writing—Original Draft*, S.K., Y.Y.O., L.K., A.E.E.; *Project Administration*, Y.Y.O., A.E., A.E.E.; *Validation*, L.K., A.E.E.; *Supervision*, A.E.E.; *Writing—Review and Editing*, A.E.E.

## ETHICAL STATEMENT

Written informed consent was obtained from the patient prior to publication.

## Data Availability

The data that support the findings of this article are available on request from the corresponding author. The data are not publicly available due to privacy and ethical restrictions.
